# Implementation and Long-Term Outcomes of Organisational Health Literacy Interventions in Ireland and The Netherlands: A Longitudinal Mixed-Methods Study

**DOI:** 10.3390/ijerph16234812

**Published:** 2019-11-29

**Authors:** Marise Kaper, Jane Sixsmith, Louise Meijering, Janine Vervoordeldonk, Priscilla Doyle, Margaret M. Barry, Andrea F. de Winter, Sijmen A. Reijneveld

**Affiliations:** 1 Department of Health Sciences, University Medical Center Groningen, University of Groningen, Hanzeplein 1, P.O. Box 30.001, FA10, 9700 RB Groningen, The Netherlands; a.f.de.winter@umcg.nl (A.F.d.W.); s.a.reijneveld@umcg.nl (S.A.R.); 2 Health Promotion Research Centre, National University of Ireland Galway, University Road, H91 TK33 Galway, Ireland; jane.sixsmith@nuigalway.ie (J.S.); priscilla.doyle@nuigalway.ie (P.D.); margaret.barry@nuigalway.ie (M.M.B.); 3 Population Research Center, Urban and Regional Studies Institute, University of Groningen, Landleven 1, P.O. Box 800, 9700 AV Groningen, The Netherlands; l.b.meijering@rug.nl; 4 Health Impact Gezondheid & Zorg, Blinkertlaan 7, Dishoek, 4371 PV Veere, The Netherlands; info@healthimpact.nl

**Keywords:** organisational health literacy, health care organisation, implementation, health equity, communication

## Abstract

Organisational Health Literacy (OHL)-interventions are needed to overcome health inequality. OHL-interventions have successfully identified communication barriers at the organisational level, but evidence is limited on the extent to which this leads to sustainable organisational change. This study aims to assess the implementation fidelity, moderators (barriers and facilitators), and long-term impact of OHL-interventions in hospitals in Ireland and The Netherlands. We used a longitudinal mixed-methods approach to assess two similar OHL-interventions in one Irish and three Dutch hospitals. The OHL-interventions concerned the improvement of navigation and implementation of health literacy-friendly communication throughout organisations. Participants were 24 hospital employees and 40 older adults who use hospital services. At six, eight, and eighteen months, we assessed the level of implementation, barriers and facilitators, and impact through questionnaires and in-depth semi-structured interviews. After older adults and professionals had identified a number of communication problems, we found that professionals had successfully implemented OHL-interventions to promote navigation and comprehensible communication. Limited resources and variation in organisational structures and procedures were perceived as barriers to implementation. The participation of service users, leadership support, and a stepwise implementation of interventions were perceived to facilitate implementation. In the long term, the OHL-interventions led to system-wide improvements, as shown by better embedding of health literacy policies, enhanced patient engagement, provision of plain language training and comprehensible information. Findings were similar for the two countries. Embedded OHL-interventions resulted in sustainable and system-wide health literacy changes in all four hospitals. Following implementation, OHL-interventions have the potential to promote health equity and empowerment among health service users.

## 1. Introduction

For people with limited health literacy, health care organisations are often complex to access and to navigate, and people encounter difficulties in understanding the information provided [1,2,3]. As almost half of the European population is reported to have inadequate health literacy, i.e., the skills to access, understand, appraise and communicate about health information [4], these problems contribute considerably to inequality, poorer health outcomes, more frequent use of emergency care and hospitalisation [5]. Although health literacy problems originate partially from individual abilities, these problems can be augmented by the growing demands of navigating complex health care systems and by the increasing responsibility of people to engage in their own health care [6]. Several studies have emphasised that healthcare organisations should enhance the accessibility and comprehensibility of information to improve patient safety and quality of care [7,8,9]. A systems approach is needed to better align the health care demands with people’s abilities and mitigate the impact of health literacy problems [6,8].

In order to reduce the level of health literacy required, health care organisations can make it easier for people to navigate health services and understand and use health information to take care of their own health [8]. Several studies have reported changes on aspects that are of major importance to become a health-literate organisation [8,10,11,12,13], which, for example, relates to leadership, systems and policies, accessible services, and effective communication. Leadership is important for integrating health literacy into the mission and culture of an organisation. Changing systems, processes, and policies may be required to ensure effective care for low health-literate people. Capacity building of employees may be needed to be health literacy responsive. Other health literacy aspects regard accessible services and programs for all service users, use of effective health literacy communication strategies, comprehensible information that is easy to understand and act on, and engagement of service users. External facilitators for health-literate organisations may be health literacy policy guidelines and funding provided by government organisations.

Organisational Health Literacy (OHL)-interventions have emerged as a promising strategy to work on health literacy responsive organisations, and involve assessment of communication barriers, as well as implementation of changes at various organisational levels [12,13]. OHL-interventions have been successfully implemented in various health care settings, such as hospitals, primary care settings, pharmacies, and other community settings, including municipalities [3,14,15,16,17]. OHL-interventions often include multiple components to assess the awareness of health literacy, the quality and comprehensibility of digital and printed information, to enhance verbal communication in patient-provider interactions, and the physical navigation of the hospital environment [3,16,18,19].

Two reviews reported that although OHL-interventions are commonly used to address organisational health literacy, their evidence is limited with respect to the generation of organisational changes and system-wide embedding of health literacy friendly communication [12,13]. Several studies found mixed results during the development of OHL-interventions and implementation periods of up to six months [3,12,13,14,19,20,21]. Some studies have reported positive experiences with OHL-interventions, facilitators for implementation, and outcomes of increased health literacy awareness and some preliminary improvements regarding the comprehensibility of information [3,12,13,14,19,20,21]. Facilitators promoting implementation were, for example, generating enthusiasm among professionals, management support, and adaptability of OHL-interventions [16,18,19]. However, other studies reported on barriers encountered during implementation and found that sustainable organisational changes were hard to realise [12,13,21,22]. Barriers to implementation of OHL-interventions included lack of leadership support, limited knowledge of quality improvement, lack of resources, and activities interfering with other work related demands [15,16,19,23]. Implementation barriers and the relative brief implementation period of up to six months could have influenced planning decisions and may have prevented observation of longer term effects [23,24]. Recently, a few studies reported positive outcomes in various settings when the implementation of OHL-interventions was guided by a systematic approach and longer implementation periods of up to three years [17,25,26]. These studies reported successes such as organisation-wide integration of health literacy into health systems and into processes for quality improvement, extensive application of health literacy interventions, increased competencies of staff, and improved understanding and actionability of patient-information materials [17,25,26].

It is clear from extant studies that the implementation of OHL-interventions to work on health literacy responsive organisations requires many steps and the underlying processes facilitating or hindering implementation may be complex. To date, few studies have systematically focused on the implementation fidelity of OHL-interventions in the longer-term. Implementation fidelity refers to the degree to which an intervention is delivered as intended [16,27,28]. Studying implementation fidelity of OHL-interventions, their moderators, including barriers and facilitators affecting implementation, and long-term outcomes, are essential for healthcare organisations to decide on the introduction of new approaches and policies to improve the accessibility and quality of care for patients with low health literacy. The aim of this study is to assess the implementation fidelity, moderators (barriers and facilitators), and the long-term impact of OHL-interventions in hospitals in Ireland and The Netherlands.

## 2. Materials and Methods 

### 2.1. Design

**Figure 1:**
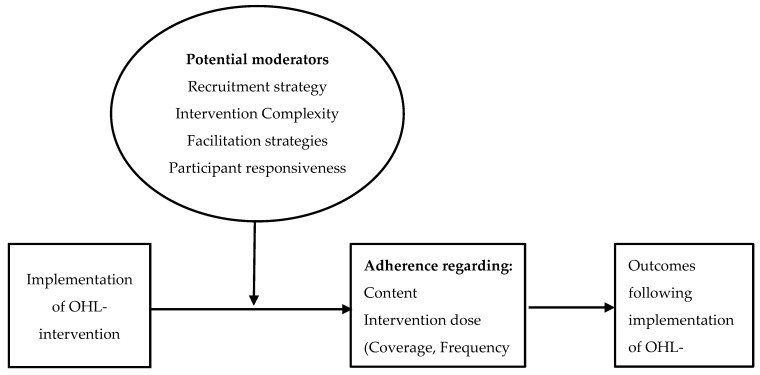
The Implementation Fidelity Model [27,28] used to assess the implementation of Organisational Health Literacy (OHL)-Interventions.

We used a longitudinal mixed-methods approach to assess the implementation fidelity of two similar OHL-interventions in one Irish and three Dutch hospitals. OHL-interventions can be classified as “complex structural interventions” as they address barriers in the “real world” healthcare context through interaction with stakeholders in the organisation [29,30]. From January 2015 to June 2016, we implemented two OHL-interventions with similar aims and components, one being developed in Ireland and the other in The Netherlands. We assessed the implementation of OHL-interventions using a comprehensive Implementation Fidelity Model [27,28], see Figure 1. 

The implementation of both OHL-interventions involved three stages (Table 1). We assessed implementation fidelity, moderators, and the impact of OHL-interventions [27] at the end of each stage using questionnaires and semi-structured interviews.

### 2.2. Setting and Sample

Four hospitals were recruited by means of convenience sampling: In Ireland, this was one teaching hospital (setting 1), and in The Netherlands, these were one rehabilitation centre being part of a larger teaching hospital (setting 2), and two hospitals (setting 3 and 4).

At the start, implementation coordinators and other members of project committees were assigned to coordinate the implementation process in each setting. Implementation coordinators were two senior nurses in setting 1; the first had a patient liaison remit, and the second was an officer working in health promotion. Coordinators were a policy advisor and a communications officer in setting 2, and two communication consultants in setting 3 and 4, respectively. Project committees consisted of ten members in setting 1 (IRL), five in setting 2, eight in setting 3, and only one implementation coordinator in setting 4 (because resources and management commitment were limited). Committee members worked on various hierarchical levels, and in different roles. Professionals involved were managers and team leaders (middle management level) in setting 1, 2 and 3, communication consultants in all settings, and senior nurses in setting 1 and 3. Professionals for the project committees were invited by the hospital executive boards. Members of the executive boards did not take part in the committees. Project committees approached other professionals who participated in the OHL-assessment. In order to assess implementation, the researchers collected data among implementation coordinators, members of project committees and other professionals, such as managers, nurses, and staff from communications and other disciplines, such as information technology and strategic management. The study was conducted in accordance with the Declaration of Helsinki and appropriate ethical guidelines in each country. In Ireland, the study-protocol was approved by a university Research Ethics Committee (reference number 14/NOV/06) in December 2014 and exempted from further independent ethical review by the University Medical Center Groningen, Medical Ethical Committee (registration number 2016/909) in The Netherlands. Participants in both jurisdictions provided written informed consent. 

### 2.3. Implementation of the OHL-Interventions

First, we provide a description of the Irish and Dutch OHL-interventions and second, we describe for each implementation stage how project committees worked on the implementation of both interventions. Section 2.4 contains a description of the measurement instruments used by the research teams in Ireland and The Netherlands to assess implementation fidelity at the end of each implementation stage.

Both the Irish intervention, “The Literacy Audit for Healthcare Settings” toolkit [31] and the Dutch intervention “Quickscan Health Literacy Toolbox” [32] were selected by the research teams. Both interventions were among the few comprehensive OHL-interventions developed within their specific country context and considered suitable to be used in a hospital setting.

#### 2.3.1. Description of the Two OHL-Interventions

1. The Irish intervention, “The Literacy Audit for Healthcare Settings” toolkit [31], included guidelines on health literacy friendly communication, and an OHL-assessment tool to assess communication barriers. The core OHL-assessment involves 57 questions, which are completed by professionals on paper about awareness of health literacy, signs and other devices for navigating premises, digital and print materials, and verbal communication. In addition, the OHL-intervention includes references to best practices in health literacy for organisations, such as the use of visuals in written materials and the ways to present numbers. The CDC Clear Communication Index [33] aligned with these requirements and was used to review written materials. In addition, the intervention refers to walking interviews [34] together with service users in order to assess the navigation from their perspective. The intervention was informed by literature for health literacy communication and is freely available online [31].
2. In The Netherlands, the Quickscan Health Literacy Toolbox [32] included information on health literacy and organisational change processes, and an OHL-assessment tool to assess the components of navigation, oral communication, written print materials and digital communication, and an action planning format for implementation. Each OHL-assessment component consisted of a self-evaluation checklist for professionals and guides for observing and/or interviewing service users. The intervention was informed by a literature review on health literacy communication and a pilot-test in various Dutch hospitals [32,35,36].

#### 2.3.2. Implementation of OHL-Interventions

##### Stage 1. Planning and Assessment

At the start, an introduction meeting was organised with the project committees in all settings. The researchers introduced the concept of health literacy, explained the OHL-intervention, the implementation stages, and how to recruit other professionals and older service users to take part in the OHL-assessment. Researchers also introduced the study and explained the measurement instruments to assess implementation fidelity.

In the first six months, the project committees in each setting planned and conducted the OHL-assessment, and recruited other professionals and health service users who took part in the OHL-assessment. In order to conduct the OHL-assessment, the Irish professionals used the Literacy Audit for Healthcare Settings, toolkit [31], and professionals in the three Dutch hospitals used the Quickscan Health Literacy Toolbox [32]. In each setting, the project committees could choose the components of the OHL-assessment they wanted to assess, which were:

- Navigation and signage within health settings
- Interpersonal communication
- Written print material
- Digital content on websites.

In order to assess OHL from the perspective of service users, the project committee in each setting recruited potential older service users with low health literacy through adult literacy services and invited older service users, who were assumed to have higher health literacy levels through convenience sampling. Researchers supported the project committees in the recruitment. Before the OHL-assessment, older service users filled in questions about their age, educational level, current health status, living situation and three self-report questions on health literacy, as defined by Chew et al. [37] (for example: “how often do you have problems learning about your medical condition”). In total, 40 service users (Ireland n = 20, The Netherlands n = 20) actively participated in the identification of communication barriers by reviewing written print materials, using websites, and navigating through organisations, while being observed and/or interviewed by professionals.

##### Stage 2. Action Planning

In month 7 to 8, the project committees analysed the results of the OHL-assessment. They calculated which communication barriers were most frequently encountered and described the themes and barriers identified by health service users. Next, project committees applied the action-planning format. Based on the assessment outcomes, project committees discussed which actions should be prioritised and planned in order to improve the communication barriers encountered. 

##### Stage 3. Implementation of Actions Planned

During month 9 to 18, project committees worked on the implementation of the actions planned. They tailored the implementation of their actions to the developments and context of their setting. Committees worked, for example, on a health literacy policy and/or the improvement of outpatient letters.

### 2.4. Procedure and Measures

At the end of each implementation stage, the researchers measured the implementation fidelity, the moderators, and the outcomes regarding the impact of OHL-interventions. We first define the six domains following The Implementation Fidelity Model [27,28], and then describe the research measurements for each implementation stage.

First, “Implementation fidelity” was defined as the delivery of OHL-interventions as intended, related to the full content of an intervention and intervention dose (being the frequency, duration and coverage of an intervention) [27]. Second, moderators were measured on six domains that can affect implementation as a facilitator or a barrier [27,28,38]. The six domains included (a) “Recruitment of participants”, which related to the consistency of procedures followed, and specific subgroups included. (b) “Intervention complexity” related to clarity of instructions and complexity of OHL-interventions. (c) “Facilitation strategies” in order to optimise fidelity, such as training or feedback. (d) “Participant responsiveness” was the engagement of professionals and service users with OHL-interventions. (e) “Quality of delivery” refers to the dedication of the professionals who are responsible for the implementation of the OHL-intervention. (f) “Context” related to social systems, structures and cultures of organisations, and inter-organisational linkages. Third, outcomes regarding “the impact of OHL-interventions” were defined as organisational changes to help people with low health literacy to navigate services, and understand and use information to maintain their health [8]. 

#### 2.4.1. Stage 1

Professionals completed a questionnaire after the OHL-assessment containing two sections: (1) To assess implementation fidelity, the researchers asked which OHL-components professionals had assessed, and how much knowledge and experience they had with applying these kinds of interventions; (2) the professionals reported how the moderators of “participant responsiveness and intervention complexity” could have affected the OHL-assessment (Supplementary Materials, Table S1). Questions employed a 5-point Likert-type scale (1 = totally disagree to 5 = totally agree).

#### 2.4.2. Stage 2

The questionnaire consisted of three sections. Implementation coordinators completed section one (on the OHL-assessment and action plan) and two (on implementation fidelity), with support from the researchers. Members of the project committee completed section three (on moderators). In the first section coordinators reported which components of OHL interventions they had assessed, which forms they had used (i.e., checklists, interviews or observation), and in which format (on paper or online) and which actions the committees had planned. Coordinators also reported on the recruitment of professionals and older service users. In the second section, coordinators reported on implementation fidelity: To what degree the components were adapted with respect to content and intervention dose. In section three, professionals in the project committee reported on the perceived moderators (intervention complexity, facilitating strategies, participant responsiveness, quality of delivery, context), see Supplementary Materials, Tables S2–S6. Questions employed a 5-point scale (1 = strongly disagree to 5 = strongly agree). 

The researchers conducted semi-structured interviews to explore in-depth perspectives of the project committee members. Interviews involved three topics. (1) The implementation fidelity of the OHL-assessment and action plan. (2) The influence of the moderators (recruitment, intervention complexity, facilitating strategies, participant responsiveness, quality of delivery, and context). (3) The intermediate outcomes of the OHL-assessment and the actions planned to improve communication and reduce barriers. All interviews with professionals lasted between 30 and 90 min and were recorded and transcribed verbatim in the local language.

#### 2.4.3. Stage 3

Researcher MK conducted follow-up interviews to assess the perspectives of the implementation coordinators and professionals directly responsible for implementation, in order to get a clear overview of actions undertaken at 18 months follow-up. In follow-up interviews the following topics were explored: (1) implementation fidelity to actions originally planned and undertaken; (2) perceived influence of moderators (recruitment, intervention complexity, facilitating strategies, participant responsiveness, quality of delivery, and context), and (3) outcomes with regard to actions undertaken in order to evaluate the impact of the OHL-interventions. 

### 2.5. Analysis

We first described organisation and participant characteristics based on the questionnaires. Next, we performed a qualitative content analysis of the interview data from stage 2 and 3 in three steps to enhance inter-coder reliability [39]. First, the researchers MK, JS, and LM jointly created an English language-coding scheme, with themes and codes derived from Carroll’s model (Figure 1). Second, two researchers independently coded two interview transcripts from each country. Researchers discussed differences and reached a consensus on a final coding scheme. As a third step, MK coded all transcriptions and linked the codes to the overarching themes originating from The Implementation fidelity Model [27,28]. The researchers MK, LM, and JS reviewed and reached a consensus on the themes, after which we reported on the findings related to implementation fidelity, moderators and outcomes, see Figure 1 [27,28].

## 3. Results

### 3.1. Organisation and Participant Characteristics

Four hospitals and twenty-four professionals in Ireland and The Netherlands participated in the study. Hospitals varied with respect to the number of professionals, beds, and healthcare provided (Table 2).

### 3.2. Implementation Fidelity

Regarding the implementation fidelity of OHL-interventions, we report on (1) content and (2) intervention dose.

(1) Content Professionals in Irish and Dutch hospitals reported high implementation fidelity related to the components of OHL-interventions they had chosen. Professionals conducted the assessment (together with service users) and worked on the implementation of actions (Table 3).

#### 3.2.1. Stage 1. OHL Assessment

Prior to implementation, the delivery format of OHL-interventions was tailored to the specific hospital setting. For example, committees had formulated specific assignments related to the route of the walking interview or selected the most relevant leaflets for patients. All four project committees involved professionals and older service users in the assessment, involving navigation, written print materials, digital content on websites, or interpersonal communication. In setting 1, the researchers completed the OHL-assessment with service users. Some senior nurses in setting 1 had difficulty completing the assessment because they had limited knowledge of policies pertaining to the development of printed materials and websites. They, therefore, received assistance from the coordinators. In setting 2, professionals did not assess navigation because of ongoing renovation activities, and the website was not assessed, as this was part of the larger hospital website and could not be adapted. In setting 3, the committee anticipated too much resistance among professionals to assess interpersonal communication. In setting 4, one active implementation coordinator was involved, but there were few resources available to form a project committee and to assess other components, apart from the website.

#### 3.2.2. Stage 2 and 3

Dutch professionals in settings 2, 3, and 4 tailored the general action plan format to their own organisation. They felt that instructions should contain more detail on how to translate the assessment results efficiently into an action plan. In setting 2, 3, and 4, professionals worked on the implementation of actions as planned following the assessment. Originally in setting 1 and 3, professionals had explored opportunities to work on the clarity of navigation, but for several reasons, this proved difficult to work on. Professionals reported that changing the signage throughout the hospital would be complex and indicated that limited resources were available for such a project. In addition, professionals perceived that their knowledge was too limited to determine what types of signage would be understood by various patient categories. In setting 1, the project committee planned and worked on the organisation-wide improvement of written printed materials and outpatient letters and decided to work on oral communication and the website in a later stage. The impact of OHL-interventions is specified in Section 3.4.

(2) Intervention Dose (which refers to the frequency, coverage, and duration of OHL-Interventions). In general, in the four settings, the components of the OHL-intervention were implemented in line with the instructions of the intervention. Only the duration of the OHL-intervention activity took longer than anticipated. Related to the frequency of the interventions, we conclude that the OHL-components were delivered as was planned and were conducted as often as was prescribed (for example, related to the number of selected leaflets, assignments during the walking interview, and implementation of actions). Regarding the coverage of OHL–interventions, the instructions specify that both (health) professionals and service users with limited health literacy should take part in the OHL-assessment, which was the case in all four settings. Professionals indicated that participation of both professionals and service users enhanced the quality of the OHL-assessment. Service users were able to identify unique barriers related to communication and navigation and talked about the impact of the barriers. These barriers could not have been observed by single professionals, whereas staff was able to identify barriers at the organisational level. For example, staff identified structural communication barriers in written and digital materials. They reported that revising those materials would take longer because the various departments involved had different working procedures. In total, 40 service users (Ireland n = 20, The Netherlands n = 20) actively participated in the assessment and a variety of professionals facilitated implementation at different organisational levels in line with the instructions of the intervention. 

In setting 1, 20 service users assessed barriers related to navigation and reviewed leaflets and outpatient letters in two focus groups (one with participants with lower and one with participants with higher health literacy). Seven professionals filled in the general assessment tool. In setting 2, four service users reviewed a lengthy information leaflet on rehabilitation and some examples of outpatient letters and forms and commented on their experiences in general. Five professionals also evaluated this leaflet, as well as the letters and forms. Five other health professionals evaluated their interpersonal communication with patients. In setting 3, twelve service users took part in the navigation assessment, reviewed a leaflet on colonoscopy and some examples of outpatient letters and forms, and used the hospital website, while being observed by professionals. Eight professionals also evaluated the selected patient leaflets, letters and forms, as well as the website. In setting 4, eight service users, from an adult education class, used the website, which was also evaluated by six professionals.

Implementation took longer than anticipated. Professionals of all settings felt that the duration of implementation activities was not clearly described and reported having limited time to work on OHL-implementation.

### 3.3. Moderators Influencing Implementation

Both Dutch and Irish professionals reported that all six moderators [27,28] influenced the implementation of OHL-interventions either as facilitators or barriers. In relation to stage 1, the moderators, including recruitment, facilitation strategies, participant responsiveness and intervention complexity were perceived to facilitate implementation. Related to stage 2 and 3, professionals reported on the quality of delivery and identified four additional contextual moderators. Contextual barriers identified were: (1) different organisational structures and procedures and (2) limited resources. Contextual facilitators identified were: (3) embedding OHL-interventions into ongoing activities, (4) obtaining leadership support. Other perceived facilitators were the participation of service users and stepwise implementation of interventions, which increased the quality of delivery.

We summarise the findings from the interviews and questionnaires and present illustrative quotes in Table 4. The tables with the descriptive findings of the questionnaires are presented in the online Supplementary Files.

#### 3.3.1. Stage 1

Recruitment: Dutch and Irish professionals in all settings reported that service users helped to identify unique communication barriers, which were not observed by the professionals themselves (e.g., the service users did not scroll on websites). These were eye-openers for professionals and highlighted the relevance of the OHL-intervention to overcoming communication barriers. In all settings, service users with low health literacy were invited sensitively to avoid generating potential feelings of shame. Although cooperation with adult literacy services facilitated the recruitment of service users in settings 1, 3, and 4, professionals experienced that the recruitment of service users required a lot of time. During the assessment conducted jointly with service users, professionals formulated concrete questions and took extra time for explanation. Professionals of the project committees in settings 1, 2, and 3, also valued the cooperation with various colleagues, which, they felt, made it easier to embed activities related to OHL-implementation at various levels in the organisation. In particular, working on the revision of outpatient letters, leaflets, and providing plain language training, required cooperation from managerial level to front line professionals. 

Facilitation strategies: Professionals reported several facilitation strategies to enhance implementation fidelity in both countries. Support from external researchers and facilitators empowered professionals in settings 1, 2, and 3. The external support contributed to their knowledge of health literacy and added credibility to the implementation of the OHL-intervention. In the questionnaires, professionals in settings 1, 2, and 3 agreed that the introduction meetings and feedback by researchers were clear, useful and sufficient. In interviews, professionals reported that the introduction meetings helped to get a better understanding of the OHL-intervention.

Participant responsiveness: Dutch and Irish professionals in all settings reported commitment to implementation, as they found OHL-interventions to be helpful in reducing communication barriers. Professionals were particularly enthusiastic about the joint assessment with service users, as they identified unique barriers related to communication and navigation. Service users raised a lot of awareness among professionals, as they not only demonstrated where more plain language should be used but also how they experienced the impact of the communication barriers encountered. The majority of professionals reported satisfaction with the OHL-interventions in the questionnaires. 

Intervention complexity: Professionals in all settings reported the implementation of OHL-interventions to be easy, straightforward, and comprehensive, but also resource-intensive to undertake. For example, the checklists for reviewing websites or written materials were perceived as clear. In addition, instructions to conduct the assessment with service users were straightforward, although some experience with comprehensible communication strategies in conversations with low-literate people was required. However, professionals reported it was resource-intensive to plan the assessment activities, to recruit service users and to analyse and present the results from the OHL- assessment. To facilitate wider application, professionals in the Dutch settings 2, 3, and 4, therefore, preferred a more concise, user-friendly, and online intervention format for the assessment and the action plan

#### 3.3.2. Stage 2 and 3

Quality of delivery: Professionals in settings 1, 2, and 3 reported implementing interventions in a stepwise fashion, starting with changes that were easy to realise. Over time, professionals reported that small pilot activities contributed to broader quality improvements. For example, an assessment of selected leaflets and outpatient letters contributed to plain language guidelines, plain language trainings, and improved patient leaflets and outpatient letters throughout settings 1, 2, and 3. Components were not implemented when professionals had limited influence on the process. For example, professionals in setting 2 did not assess navigation because of ongoing renovation activities in their location. In addition, professionals in setting 3 were concerned that the assessment of communication skills would generate resistance, as health professionals considered their own communication skills to be of good quality already. 

Context: Professionals reported on four specific context related moderators, two of which were barriers to implementation. (1) Different organisational structures and procedures made implementation more complex, for example, departments in settings 1 and 3 had different procedures for producing leaflets and patient letters, which made revisions more difficult and required cooperation at various organisational levels. (2) Limited resources protracted implementation activities in all settings. Professionals reported that, in general, the time, money, and workforce to undertake OHL-intervention activities were limited. Despite this, the anticipation of professionals on two other contextual moderators facilitated implementation. (1) Professionals embedding OHL-interventions into ongoing activities fostered implementation and improved its position on the management agenda. (2) Obtaining leadership support from various organisational levels was considered as essential by professionals to prioritise implementation and allocation of resources. 

### 3.4. Impact of OHL-Interventions

The implementation resulted in a range of outcomes aimed at supporting people with low health literacy (Table 5). After the OHL-assessment in stage 1, professionals in all four settings reported greater awareness of low health literacy and related communication barriers, particularly regarding the difficulties of service users with scrolling or using search functions on websites, reading, and interpreting inconsistent names and numbers on signage, and understanding the information in outpatient letters.

During stage 2 and 3, settings 1, 2, and 3 planned and embedded system-wide changes in health literacy friendly communication, such as establishing a health literacy committee, health literacy policies, plain language training, and improved communication products like leaflets, letters, and websites. In addition, setting 4 worked on further improvement of their website. Although professionals in settings 1 and 3 explored options to improve navigation, for example, through the development of a clear sitemap, this proved difficult to realise, and structural changes were not implemented. Professionals reported that changing signage would be complex to realise in the hospital, that resources would be limited, and that they had limited knowledge of what types of signage would be adequate and understood by different service users.

After eighteen months, healthcare organisations with active project committees, knowledge of implementation processes, and a well-designed action plan reported the strongest quality improvement. 

## 4. Discussion

This is the first longitudinal study to assess the degree of implementation (implementation fidelity), the barriers and facilitators, and the long-term impact of OHL-interventions in Irish and Dutch hospitals over an eighteen-month period. OHL-interventions were implemented with high fidelity. Perceived contextual barriers to implementation were “different organisational structures and procedures” and “limited resources”. Facilitators identified were “obtaining leadership support”, “embedding OHL-interventions into ongoing activities”, “active participation of service users” and “a stepwise approach to implementation” of interventions. In the long term, the OHL-interventions led to system-wide improvements, as shown by improved embedding of health literacy policies, increased patient engagement, provision of plain language training, and comprehensible written and digital information. Findings were similar for the two countries. 

In line with other studies on implementation fidelity [27,28], professionals of four healthcare organisations implemented OHL-interventions with high fidelity and tailored the format to optimise its delivery [19,23]. The components chosen most often were written and digital communication and navigation [3,14,19,23] because they were perceived as relevant and feasible to implement. One project committee chose not to assess oral communication because they anticipated resistance among health professionals. Nevertheless, other studies reported that health professionals do not always use clear communication techniques [3,14,17,19]. Brief training in health literacy awareness could help to overcome any potential resistance [14]. 

We found evidence for all moderators mentioned in The Implementation Fidelity Model [27,28], which either were facilitators or barriers to implementation. This evidence is also in line with barriers and facilitators reported in two reviews on OHL-interventions [12,13]. In particular, active participation of service users facilitated implementation and engaged professionals, because service users identified barriers not observed by professionals. Although in other studies of OHL-interventions where service users were interviewed [14,20,24], none reported stronger awareness of health professionals of barriers encountered by users. Our findings suggest that the involvement of service users is essential in the assessment of barriers. The responsiveness of professionals and facilitation strategies, such as feedback and support from external researchers, also promoted implementation [19,23,27,40]. 

Like Hasson et al. [28,40], we identified four specific contextual moderators, but a distinct finding of our study was that professionals anticipated the influence of context. Limited resources and variation in organisational structures and procedures were barriers to implementation fidelity [19,23,40]. Professionals anticipated solving these contextual barriers by obtaining leadership support to prioritise implementation, allocate resources, and embed OHL-interventions into ongoing activities. 

Our study showed that in various settings, system-wide improvements, such as health literacy policies, plain language training, and comprehensible written and digital information, were embedded at various organisational levels, which is in line with a few other recent studies using more systematic implementation approaches over longer periods [17,25,26]. Two studies carried out at the same site [25,26] reported successes, such as integrating health literacy into health systems and quality improvement processes, and creating a governance structure and a web-based platform for developing and testing plain health information [25]. This resulted in organisation-wide improvement of patient information materials related to their understanding and actionability [26]. Another study [17] reported embedding a variety of locally relevant health literacy interventions at nine sites, which resulted in process improvements at the organisational level, improved knowledge and skills among staff, community engagement, and some improvement in client outcomes. This finding contrasts with those studies on OHL-intervention implementation that cover shorter periods [12,13,18,19,20,24]. Those studies reported the identification of communication barriers and preliminary improvements because time, knowledge on how to implement health literacy practices, and resources were limited [12,13,18,19,20,24]. In our study, both Irish and Dutch professionals reported that a pilot assessment sufficed to identify relevant deficits for the entire organisation. This widened the scope for quality improvements, particularly if a project committee had knowledge of implementation processes and engaged in a stepwise implementation of the OHL-interventions. However, navigation by patients did not improve as professionals reported that resources were insufficient. In addition, similar to other studies [3], professionals acknowledged the navigation barriers faced by service users and indicated that navigation should be considered when designing or renovating health facilities.

To summarise, this evaluation of the implementation of OHL-interventions provides insight into the health literacy barriers encountered, but also in the processes that generate organisational change for embedding health literacy friendly communication and patient-centred care [13]. Stepwise implementation of these interventions resulted in system-wide improvements in the long-term. 

### 4.1. Strengths and Weaknesses

This study has several strengths. We used a comprehensive implementation framework [27,28,41] and a long-term period to assess implementation fidelity, moderators, and outcomes of interventions. Interviews and questionnaires complemented each other in collecting data on perspectives from a variety of professionals involved in four different healthcare organisations in two countries. This study also has some limitations. The self-report questionnaires and interviews we used to study professionals’ perceptions may have been influenced by social desirability and recall bias. Furthermore, we did not investigate outcomes among service users after implementation, although we found that the assessment of information by service users contributed to quality improvements in written and digital communication products. 

### 4.2. Implications

This study shows that OHL-interventions need to implement those components that are most relevant to specific healthcare organisations and that have the greatest impact on the quality of health care. As the majority of studies on OHL-interventions originate from the United States [12], the similarity of our findings in two European countries is promising for implementation of OHL-interventions in other healthcare organisations in Europe. Our findings inform the development of future OHL-interventions and programs that focus on implementation and organisational change in order to develop health-literate health care organisations. In the longer term, implementation of OHL-interventions can contribute to health literacy-friendly organisations. Governmental support, for example, by initiating policies, interventions and funding, can stimulate a systems-approach to organisational health literacy in order to reduce health inequality and the impact of health literacy related problems [10,42]. The next steps for future research are to evaluate the impact of OHL-implementation on the quality of healthcare, communication with patients, and their health literacy levels. 

## 5. Conclusions

This evaluation of the implementation of OHL-interventions provides insights into how to generate organisational change that will promote health care organisations in becoming more health-literate and patient-centred [13]. Across four hospitals in two countries, we found that professionals implemented OHL-interventions with high fidelity to promote improved access, navigation, and comprehensible communication. Limited resources and variation in organisational structures and procedures were perceived as barriers for implementation. The participation of service users, leadership support, and stepwise implementation of interventions were perceived to facilitate implementation. In the long-term, the OHL-interventions led to system-wide improvements across the hospitals in both countries. This was shown by an improved embedding of health literacy policies, increased patient engagement, provision of plain language training, and comprehensible information. Implementation of OHL-interventions can contribute to health equity and empowerment among patients.

## Figures and Tables

**Table 1:** Overview of implementation stages, implementation activities related to the Organisational Health Literacy Interventions, and research measurements.

Implementation Stage	Implementation Activities of OHL-Intervention in Hospitals	Research Measurements
1. Planning and assessment (month 0–6)	1. Project planning and conducting of OHL-assessment	1. Questionnaire (after 6 months)
2. Action planning(month 7–8)	2. Action planning based on OHL-assessment outcomes	2. Questionnaire and interviews with project committees(after 8 months)
3. Implementation of actions (month 9–18)	3. Implementation of the actions to improve communication	3. Interviews with implementation coordinators (after 18 months)

**Table 2:** Characteristics of participating hospitals and professionals.

Characteristics of the Hospital Setting	Setting 1 (IRL ^1^) Teaching Hospital	Setting 2 (NLD ^2^) Academic Rehabilitation Centre	Setting 3 (NLD) General Hospital	Setting 4 (NLD) General Hospital
Number of professionals (fulltime equivalent)	2500	450	680	2670
Number of beds	540	120	313	468
Questionnaire N in stage 1 (professionals involved in OHL-assessment)	7	5	8	1
Questionnaire N in stage 2 (project committee).	5	4	3	1
Interviews N in stage 2 (project committee)	10	5	5	1
Interviews N in stage 3 (implementation coordinators)	2	2	1	1
Professional disciplines (project committee)				
- Management	1	1	2	-
- (Senior) nurses	4	-	3	-
- Clinical nurses	3	-	1	-
- Communication staff	1	2	1	1
- Other professionals (IT, policy, etc.)	1	2	1	-

^1^ Ireland is abbreviated as IRL. ^2^ The Netherlands is abbreviated as NLD.

**Table 3:** Implementation fidelity of the two Organisational Health Literacy (OHL)-Interventions.

	Navigation and Signage		Interpersonal Communication		Written Print Material		Digital Content	
	Assessment	Actions	Assessment	Actions	Assessment	Actions	Assessment	Actions
Setting 1	X ^1^	-	X	-	X	X	X	-
Setting 2	-	-	X	X	X	X	-	-
Setting 3	X	-	-	-	X	X	X	X
Setting 4	-	-	-	-	-	-	X	X

^1^ An X indicates the activity was implemented, an-indicates the activity was not implemented.

**Table 4:** Quotes illustrating the influence of moderators on implementation of OHL-interventions in Irish and Dutch healthcare settings.

Moderators	Illustrative Quotes from Study Respondents
**Stage 1** Recruitment	*“Ehm what applies to us is that it is filled in by very different people, from very different functions. And that you just do not have the opportunity in your daily work to get these people together in this way about one and the same subject”.* (NLD ^1^ setting 2, participant 5, interview)
Facilitation strategies	*“… and if you want you can get everything from there (from the internet) and implement it yourself but in practice it is much more awkward. Of course, it often does not work that way. And that is purely because you need a coordinator, and sometimes you will need an authority that is qualified, shall we say. Ehm that guides you or assigns the right people”.* (NLD setting 3, participant 12, interview).
Intervention complexity	*“Well, my experience is that it is a lot. That I, well yeah, that it would be nicer if it [the tool] were digitally better available. And those questionnaires were more simplified and a final results tool was added so that you could process your results more easily”.* (NLD setting 3, participant 13, interview)
Participant responsiveness	*“I think it’s been very useful, great to focus our attention on health literacy even though we knew there was work needed to be done about it. I suppose it kind of focused us and gave us a bit of momentum to get working on it. It was very practical and very clear. Everybody is very interested in it”.* (IRL ^2^, setting 1, participant 21, interview)
**Stage 2 and 3** Quality of delivery	*“Of course we have the results from the Quickscan. And if you approach that very narrowly, you look very closely at the three leaflets we have scanned and the two letters and the oral checklists […]. But the effect of applying the Quickscan is that we simply see what is actually needed. We see that many things are going well, but that there are also points for improvement in this area. In fact, we want to embed that within the center”.* (NLD, setting 2, participant 1, interview)
Contextual moderators:	
(1) Different organisational structures and procedures	*“We are a centre but we have different departments. All different departments have different methods. So when we say this must be done differently, that could affect all kinds of different working systems. […] Sometimes it is bound to a computer system that we use”.* (NLD, setting 2, participant 1, interview)
(2) Limited resources	*“So this area of stuff is seen to be like that, it will cost money and it’s letting the dust settle, letting the sun dawn on that last chapter and that we can go at it again but it really has put projects like this, stopped them in their tracks. But I think if it’s done on a phased basis where it’s broken up into smaller mini projects I think it has a lot better chance of seeing the light of day again”.* (IRL, setting 1, participant 18, interview)
(3) Embedding OHL-interventions into ongoing activities	*“See where you can reinforce each other in this area. If you are going to do something as a stand-alone project, it can be done of course, but I think that has less chance of success. I think it is nice that you link it to, there is of course a lot of attention for self-management, of course, and it links to patient-centeredness. Hospitality, so it links to so many parts. And whatever you link it to; it will give you more opportunities to implement”.* (NLD, setting 2, participant 1, follow up interview)
(4) Obtaining leadership support	*“It’s very good! The general manager, the director of nursing, they are very ehm, you know, they are committed to this. They really are! Ehm, they feel it’s very worthwhile and like that it’s ehm, it’s a patient experience initiative as well”.* (IRL, setting 1, participant 23, follow up interview)

^1^ The Netherlands is abbreviated as NLD. ^2^ Ireland is abbreviated as IRL.

**Table 5:** Quotes illustrating outcomes of OHL-interventions reported in Irish and Dutch healthcare settings.

**Stage 1. Outcomes of the OHL-Intervention Assessment**	**Illustrative Quotes**
Awareness of health literacy and related communication barriers (all settings).	*“So it has made me stop, take off my work hat and […] look at it from a patient’s perspective. So it’s made me more aware and it’s made me very anxious to try and be part of doing something about it”.* (IRL ^1^, setting 1, participant 22, interview)
Written communication (settings 1, 2, and 3): - Information and structure unclear and difficult words. - Different working procedures. - No patient centred perspective.	*“Well they (service users) were so clear about it. That they get far too much information, too many letters. And according to me, the letters also very often (contain) information that does not reach the customer at the right time, and contradictory messages in a letter and ehm well all sorts of things”.* (NLD ^2^, setting 2, participant 2, interview)
Navigation (settings 1 and 3): - Inconsistency in words and numbers on signage.	*“I mean, if you were able to come to the hospital and walk around. It can be very confusing for people. And it’s quite a large building with a lot of different areas and no two signs are the same”.* (IRL, setting 1, participant 23, follow up interview)
Oral communication (settings 1 and 2): - Information, (organisational) jargon and accents difficult to understand.	*“If we use jargon, if we choose this, you also create a distance with the patient. Besides that, it is ineffective, because he does not always understand it”.* (NLD, setting 2, participant 3, interview)
Website (settings 1, 3 and 4): - Fast reading out function, service users do not use scrolling or search function, unclear and long information.	*“Yes, because I also know that we said in advance, everyone scrolls, when you go to the website. […] Well not really. […] I saw this with those low-literate people, nobody scrolls. Everyone thinks this is it. And then there is a lot of information underneath”.* (NLD, setting 4, participant 6, interview)
**Stage 2 and 3. Organisational Changes Undertaken Following Action Plan**	**Illustrative Quotes**
Organisation wide health literacy committee established (settings 1 and 2).	*“Establishing the Health Literacy Committee was a big step. And then spreading the word about it. […] And all the hospitals in our group are very interested in it as well, ehm, because people do realise that it is very difficult for patients”.* (IRL, setting 1, participant 23, follow up interview)
Extra employee to facilitate the embedding of health literacy in working procedures and professionals’ practice (settings 2).	*“We have at least one employee for a year, so there is also a limitation in terms of employability. Hence, we also want to see as much as possible, which tools for example, we can already use for staff and administration, to train these people and provide them with skills. So that they can continue with it independently”.* (NLD, setting 2, participant 1, follow up interview)
Health literacy policy and more user-friendly checklist to assess leaflets or letters (settings 1).	*“The health literacy policy for the hospital, okay? So that people have a process to follow when they are developing not only information leaflets but I suppose any kind of (patient) information”.* (IRL, setting 1, participant 21, follow up interview)
Written communication (settings 1, 2, and 3): - Examples of comprehensible materials. - Adaption of systems to print user-friendly patient letters.	*“Ehm, yes, they obviously cannot copy sentences, because every brochure is different. But (they can take over) the tone in which a brochure is written or the layout of a text on the website. […] So that there are a number of examples that people can continue with”.* (NLD, Setting 2, Participant 5, follow up interview)
- Plain language training.	*“So they did either four hours or half a day of plain English training. […] Yeah, I think I’d say 64 or 65 people at this stage that have been trained in plain English”.* (IRL, setting 1, participant 21, follow up interview)
- Develop comprehensible materials and streamline information processes.	*“So, what we looked at was our outpatients’ letters […] we revised those letters. Now that was a process of itself and we went, we passed them with some low literacy level groups and also high literacy level groups and we’ve kind of come back to the basics of who, what, why, where and when. Yes! So we changed about three or four letters. We have over 30 letters, okay?”* (IRL, setting 1, participant 21, follow up interview)
Digital communication (websites) (settings 3 and 4): - Plain language, information reduction on webpages - Design, banners, reading function.	*“When people looked at the left in the navigation structure and when they were (looking at) a condition, they did not know exactly which treatment and what examination belonged to it. In terms of image, we made some adjustments. Another font and a different color, now it is clearer what belongs together”.* (NLD, setting 4, participant 6, follow up interview)
Navigation (settings 1 and 3)	*“Ehm, another thing we hoped to do but we didn’t get the money this time for it was a site map. You know, a simple map with all, we have quite a big site here and a lot of different buildings”.* (IRL, setting 1, participant 23, follow up interview)
Oral communication (setting 2)	*“I’m sure it will come in time […] But we definitely will look at the oral communication, but we’re not there or near to it”.* (IRL, setting 1, participant 21, follow up interview)

^1^ Ireland is abbreviated as IRL. ^2^ The Netherlands is abbreviated as NLD.

## Supplementary Materials

The following are available online at https://www.mdpi.com/1660-4601/16/23/4812/s1, Table S1. Frequency scores of moderator ‘Facilitating Strategies’ (Implementation stage 2); Table S2. Frequency scores of moderator ‘Participant Responsiveness’ (Implementation stage 1); Table S3. Frequency scores of moderator ‘Participant responsiveness’ (Implementation stage 2); Table S4. Frequency scores of moderator ‘Intervention Complexity’ (Implementation stage 2); Table S5. Frequency scores of moderator ‘Quality of delivery’ (Implementation stage 2); Table S6. Frequency scores of moderator ‘organisational context’ (Implementation stage 2).
