# Supplementary material for: Implementation and Long-Term Outcomes of Organisational Health Literacy Interventions in Ireland and The Netherlands: A Longitudinal Mixed-Methods Study
Source: Int J Environ Res Public Health. 2019 Nov 29;16(23):4812. doi: 10.3390/ijerph16234812 (PMC6926611; doi:10.3390/ijerph16234812)
Supplement: Supplementary file 1 [file ijerph-16-04812-s001.pdf]

Online Supplementary Files including descriptive results of the perceived moderators.

Table S1. Frequency scores of moderator 'Participant Responsiveness' (Implementation stage 1).

	Ireland (N = 7)				Netherlands (N = 13)			
**Moderators**	**Agree**	**Disagree**	**Neither A/D[1]**	**Missing responses**	**Agree**	**Disagree**	**Neither A/D**	**Missing responses**
**Participant responsiveness**								
- Helps identify issues in setting that may disadvantage patients	7	-	-	-	11	2	-	-
- Helps reduce time in dealing with accessibility issues and repeating information to patients	3	2	2	-	5	2	5	1
- Helps make improvements in relationship with patients	4	1	2	-	7	1	4	-
- Satisfied with intervention	3	3	1	-	9	-	4	-
- Professional obligation	5	1	1	-	6	1	6	-
- Actively involved with implementation of intervention	3	3	1	-	12	-	1	-
- I am not the correct person to implement	2	3	2	-	3	8	2	-
- Subjective norm - normative beliefs direct colleagues [2]	1	1	5	-	1	4	8	-
- Subjective norm - normative beliefs other persons [2]	1	-	6	-	3	3	7	-
- Subjective norm - motivation to comply direct colleagues and managers [3]	6	-	1	-	6	3	4	-
- Subjective norm - motivation to comply other persons [3]	7	-	-	-	9	2	1	1
- Social support	6	-	1	-	7	4	2	-
- Self-efficacy	1	4	2	-	7	1	5	-
- Knowledge	1	6	-	-	9	2	2	-
**Intervention complexity**								
- Tool is too complicated	2	5	-	-	1	10	2	-
- It is too long	3	3	1	-	2	8	2	1
- I cannot answer all questions in tool	5	2	-	-	5	7	1	-
- Provides practical way to assess health literacy	7	-	-	-	8	1	4	-

[1] Neither A/D= neither agree/disagree. [2] Alternative response scale: 1) most definitely not, 2) definitely not, 3) perhaps not, perhaps, 4) definitely, 5) most definitely, recoded into disagree (most definitely not, and definitely not), agree (definitely, and most definitely) and neither agree or disagree (perhaps not, perhaps). [3]Alternative response scale: 1)very little, 2) little, 3) not a little, not a lot, 4) a lot, 5) a great deal recoded into disagree (very little, and little) agree (a lot, and a great deal).
and neither agree or disagree (not a little, not a lot).

Table S2. Frequency scores of moderator 'Facilitating Strategies' (Implementation stage 2)

	Ireland (N = 7)					Netherlands (N = 8)				
	**Disagree**	**Neither A/D[1]**	**Agree**	**NA[2]**	**Missing responses**	**Disagree**	**Neither A/D**	**Agree**	**NA**	**Missing responses**
- Clear training/ introductory meeting	-	-	3	-	4	-	2	4	1	1
- Useful training/ introductory meeting	-	-	3	-	4	-	3	4	1	-
- Sufficient training/ introductory meeting	-	-	3	-	4	-	2	4	1	1
- Clear protocol/manual	-	-	1	-	6	3	1	4	-	-
- Useful protocol /manual	-	-	1	-	6	1	1	6	-	-
- Sufficient protocol /manual	-	-	1	-	6	3	1	4	-	-
- Clear feedback	-	-	1	-	6	-	1	4	3	-
- Useful feedback	-	-	1	-	6	-	1	4	3	-
- Detailed feedback	-	-	1	-	6	1	2	2	3	-

[1] Neither A/D= neither agree/disagree. [2] NA= if participant chose 'not applicable' or 'no' to question.

Table S3. Frequency scores of moderator 'Participant responsiveness' (Implementation stage 2).

	Ireland (N = 5)			Netherlands (N = 8)		
	Disagree	Neither A/D[1]	Agree	Disagree	Neither A/D	Agree
- Satisfied with content of OHL-intervention	-	1	4	2	2	4
- The OHL-intervention met my needs	-	2	3	1	2	5
- Engaged in the OHL-intervention	-	-	5	-	1	7
- Satisfied with delivery	-	1	4	1	1	6
- Used available resources (eg. staff with organisational competency relating to health literacy assessment)	-	1	4	-	-	8
- Recognised the benefits of OHL-intervention	-	-	5	-	1	7
- Carried out the essential activities	-	-	5	-	-	8
- Involved beyond what was expected	1	3	1	1	3	4

[1] Neither A/D= neither agree/disagree

Table S4. Frequency scores of moderator 'Intervention Complexity' (Implementation stage 2).

	Ireland (N = 5)				Netherlands (N = 8)			
	**Disagree**	**Neither A/D[2]**	**Agree**	**NA[2]**	**Disagree**	**Neither A/D**	**Agree**	**NA**
- Familiar with content	-	-	5	-	2	2	4	-
- Needed training to implement the OHL-intervention	2	1	2	-	5	2		1
- Many components to implement	-	3	2	-	3	-	5	-
- Clear descriptions	-	2	3	-	2	4	2	-
- Used to this kind of tool	4	1	-	-	6	1	1	-
- I had to perform components of the tool several times	1	1	3	-	3	1	4	-
- Detailed description of the tool sufficiently detailed	1	3	1	-	1	2	5	-
- Many difficult goals to reach	-	2	2	1	4	2	2	-
- Had to work with other professionals to deliver the OHL-intervention	-	-	5	-	1	-	7	-
- The OHL-intervention required a lot of effort from the institution/hospital setting	1	-	4	-	1	-	7	-

[1] Neither A/D= neither agree/disagree. [2] NA= if participant chose 'not applicable' or 'no' to question.

Table S5. Frequency scores of moderator 'Quality of delivery' (Implementation stage 2).

	Ireland (N = 5)					Netherlands (N = 8)				
	Disagree	Neither A/D[1]	Agree	NA[2]	Missing responses	Disagree	Neither A/D	Agree	NA	Missing responses
- The OHL-intervention would be very useful for patients	1	-	4	-	-	-	-	7	-	1
- Knew the subject of the OHL-intervention	1	1	3	-	-	1	1	5	-	1
- Used simple words	-	-	4	1	-	-	2	4	1	1
- Was highly motivated to deliver the OHL-intervention	1	1	3	-	-	-	-	6	-	2
- Repeated essential information components involving patients/clients	-	1	2	2	-	-	1	4	3	-
- Able to answer questions during implementation involving patients/ clients	1	2	1	1	-	-	-	5	3	-
- Lacked experience	-	-	5	-	-	4	3	1	-	-
- Asked open questions during implementation involving patients/clients	-	1	2	2	-	-	1	5	2	-
- Had a good knowledge of health literacy and its environmental assessment	1	2	2	-	-	-	4	4	-	-
- Actively listened to patients / clients during implementation	-	2	1	2	-	-	1	5	2	-
- Sceptical about the outcome of the OHL-intervention	3	-	2	-	-	7	1	-	-	-

[1] Neither A/D= neither agree/disagree. [2] NA= if participant chose 'not applicable' or 'no' to question.

Table S6. Frequency scores of moderator 'organisational context' (Implementation stage 2).

	Ireland (N = 4)			Netherlands (N = 8)		
	Agree	Disagree	Neither A/D[1]	Agree	Disagree	Neither A/D
**Moderators associated with the organizational context**						
- Formal ratification by management	1	3	-	-	8	-
- Replacement when staff leave	-	3	1	-	7	1
- Staff capacity	2	-	2	2	5	1
- Financial resources	-	1	3	-	4	4
- Time available	2	1	1	2	4	2
- Material resources and facilities	1	1	2	1	3	4
- Coordinator	3	1	-	5	3	-
- Unsettled organisation	2	2	-	7	1	
- Information accessible about use of the innovation	1	3	-	8	-	-
- Performance feedback	2	2	-	4	1	3
- Observability	1	2	1	3	2	3

[1] Neither A/D= neither agree/disagree
